# Retraction Note: Mixed convection flow of an electrically conducting viscoelastic fluid past a vertical nonlinearly stretching sheet

**DOI:** 10.1038/s41598-025-02616-5

**Published:** 2025-05-26

**Authors:** Ahmad Banji Jafar, Sharidan Shafie, Imran Ullah, Rabia Safdar, Wasim Jamshed, Amjad Ali Pasha, Mustafa Mutiur Rahman, Syed M. Hussain, Aysha Rehman, El Sayed M. Tag El Din, Mohamed R. Eid

**Affiliations:** 1 Department of Mathematics, Kebbi State University of Science and Technology, Aliero, P.M.B. 1144 Birnin Kebbi, Kebbi State Nigeria; 2 Present Address: Department Mathematical Sciences, Faculty Sciences, Universiti Teknologi Malaysia, Skudai, 81310 Johor Bahru, Johor Malaysia; 3 College of Civil Engineering, National University of Sciences and Technology, Islamabad, 44000 Pakistan; 4 Department of Mathematics, Lahore College for Women University, Lahore, 54000 Pakistan; 5 Department of Mathematics, Capital University of Science and Technology (CUST), Islamabad, 44000 Pakistan; 6 Aerospace Engineering Department, King Abdulaziz University, Jeddah, 21589 Saudi Arabia; 7 Mechanical Engineering Program, Physical Science and Engineering Division, King Abdullah University of Science & Technology, Thuwal, ON 23955 Saudi Arabia; 8 Department of Mathematics, Faculty of Science, Islamic University of Madinah, Medina, 42351 Saudi Arabia; 9 Department of Mathematics, University of Gujrat, Gujrat, 50700 Pakistan; 10 Electrical Engineering, Faculty of Engineering and Technology, Future University in Egypt, New Cairo, 11835 Egypt; 11 Department of Mathematics, Faculty of Science, New Valley University, Al-Kharga, 72511 Al-Wadi Al-Gadid Egypt; 12 Department of Mathematics, Faculty of Science, Northern Border University, Arar, 1321 Saudi Arabia

Retraction of: *Scientific Reports* 10.1038/s41598-022-18761-0, published online 29 August 2022

The Editors have retracted this Article.

Following the publication of this Article, concerns were raised regarding the presence of irrelevant references. Further investigation by the Editors found additional errors in the Article that include potentially problematic equations (11-14), presence of undefined variables (Rf, Rs, Sf, Ss, wg1, wg2, wg3) in the provided code that are not mentioned in the Article, as well as unverifiable authorship.

The Editors therefore have lost confidence in the integrity of this Article.

The Editors were not able to obtain the current contact details of Ahmad Banji Jafar, Syed M. Hussain, and Aysha Rehman. The remaining Authors have not responded to correspondence from the Editors about this retraction.

